# Supplementary material for: Investigation of artificial cells containing the Par system for bacterial plasmid segregation and inheritance mimicry
Source: Nat Commun. 2024 Jun 10;15:4956. doi: 10.1038/s41467-024-49412-9 (PMC11164925; doi:10.1038/s41467-024-49412-9)

Supplemental Information

Investigation of artificial cells containing the Par system for bacterial plasmid segregation and inheritance mimicry

Jingjing Zhao[1], Xiaojun Han[1]*

[1]State Key Laboratory of Urban Water Resource and Environment, School of Chemistry and Chemical Engineering, Harbin Institute of Technology, Harbin, 150001, China

*Corresponding author: hanxiaojun@hit.edu.cn (X.J.H.)

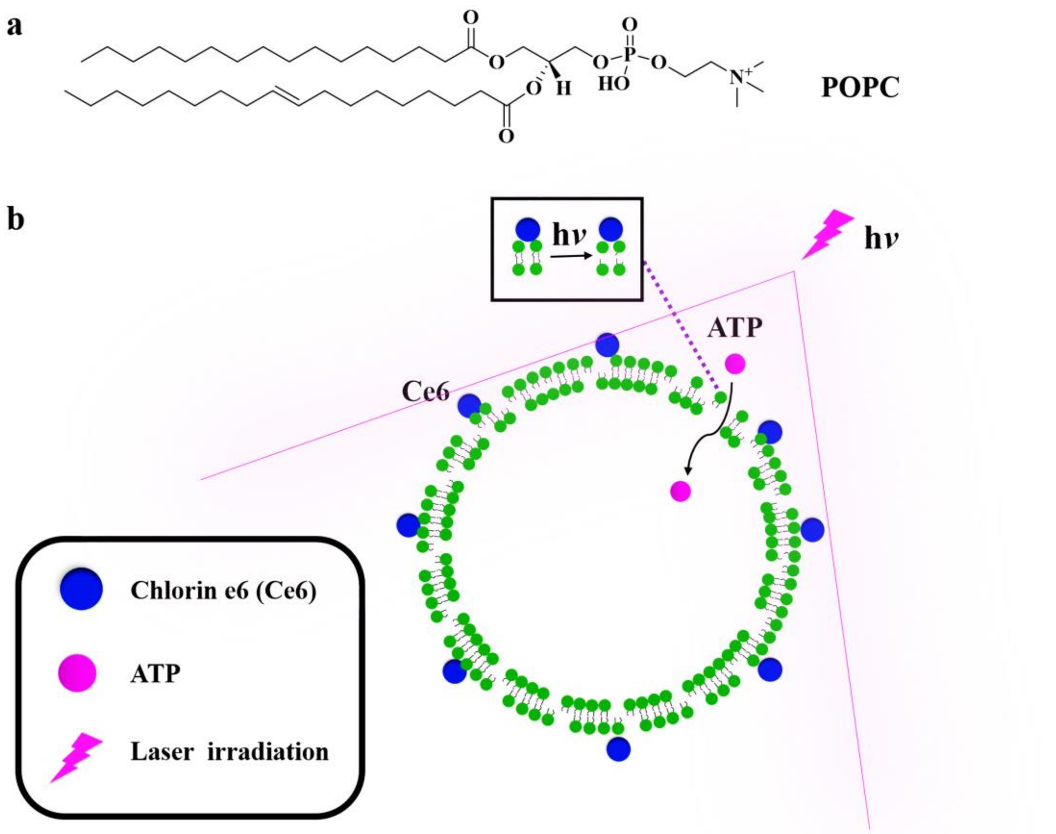

Supplementary Figure 1. The Schematic illustration of the adenosine triphosphate (ATP) inflow into giant unilamellar vesicles (GUVs) upon laser irradiation. a Molecular structure of palmitoyl oleoyl phosphatidyl choline (POPC). **b** The inflow of ATP molecules into GUVs through the transient pores due to the peroxidation of unsaturated lipids (POPC) at the existence of Chlorin e6 (Ce6) upon laser irradiation (405 nm, 0.3 mW, 5 s).

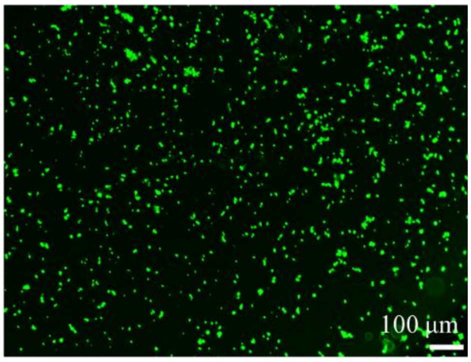

Supplementary Figure 2. Fluorescence microscopy image of the *parC*-beads. The *parC*-beads displayed green fluorescence due to the staining of *parC* DNA with SYBR Green I. The solution containing 260 mM sucrose, 30 mM Tris-HCl, 2 mM $MgCl_2$, 1 mM DTT, and 100 mM KCl at pH 7.5. The scale bar is 100 µm.

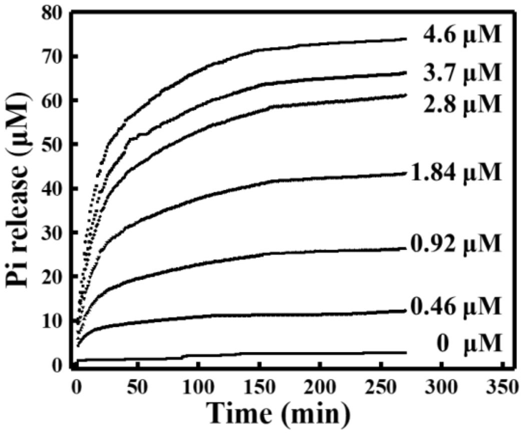

Supplementary Figure 3. Phosphate (Pi) release of adenosine triphosphate (ATP) catalyzed by ParM as a function of time. The phosphate release of ATP (4 mM) at 270 min was 2.7 μM, 12.3 μM, 26.3 μM, 43.5 μM, 61.1 μM, 66.2 μM, and 73.9 μM catalyzed by ParM with the concentrations of 0 μM, 0.46 μM, 0.92 μM, 1.84 μM, 2.8 μM, 3.7 μM, 4.6 μM, respectively. Source data are provided as a Source Data file.

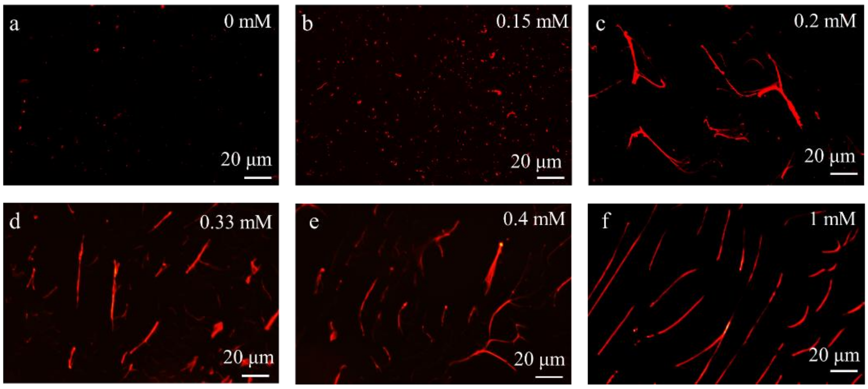

Supplementary Figure 4. Influence of adenosine triphosphate (ATP) concentration on ParM polymerization. Fluorescence microscopy images of ParM (19.1 μM) polymerization at the ATP concentrations of 0 mM (**a**), 0.15 mM (**b**), 0.20 mM (**c**), 0.33 mM (**d**), 0.40 mM (**e**), and 1 mM (**f**) within 5 min. The polymerized buffer contains 30 mM Tris-HCl, 2 mM $MgCl_2$, 1 mM DTT, 100 mM KCl at pH 7.5. The scale bars are 20 μm.

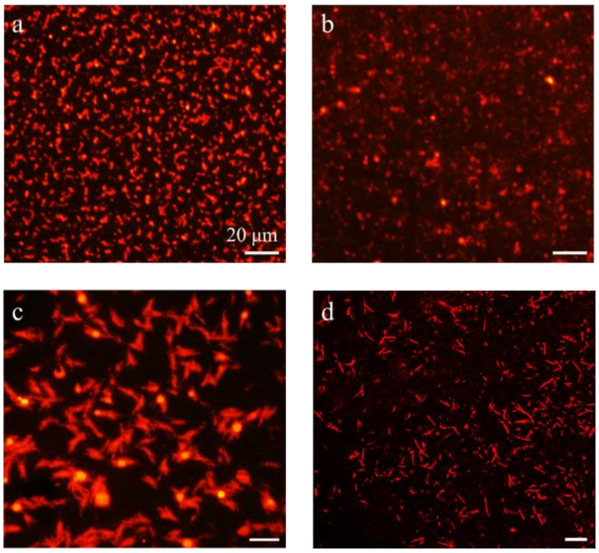

Supplementary Figure 5. The ParM polymerization behavior under different conditions. Fluorescence microscopy images of ParM (9.6 µM) filaments polymerized in the buffer solution (10 mM ATP, 30 mM Tris-HCl, 2 mM $MgCl_2$, 1 mM DTT, and 100 mM KCl at pH 7.5) containing 0.4% methylcellulose (**a**) and 0% methylcellulose (**b**). Fluorescence microscopy images of ParM (9.6 µM) filaments polymerized in the buffer solution (1 mM ATP, 30 mM Tris-HCl, 2 mM $MgCl_2$, 1 mM DTT, and 100 mM KCl at pH 7.5) containing 0.4% methylcellulose (**c**) and 0% methylcellulose (**d**). The scale bars are 20 µm.

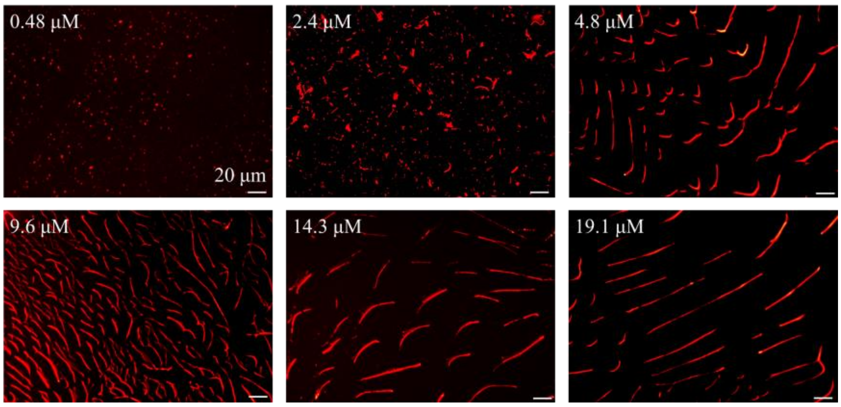

Supplementary Figure 6. Fluorescence microscopy images of ParM polymerization at different concentrations of ParM. ParM (0.48 μM, 2.4 μM, 4.8 μM, 9.6 μM,14.3 μM, 19.1 μM) was polymerized in buffer (30 mM Tris-HCl, 2 mM $MgCl_2$, 1 mM DTT, 100 mM KCl, pH 7.5) triggered with 1 mM ATP. The scale bars are 20 μm.

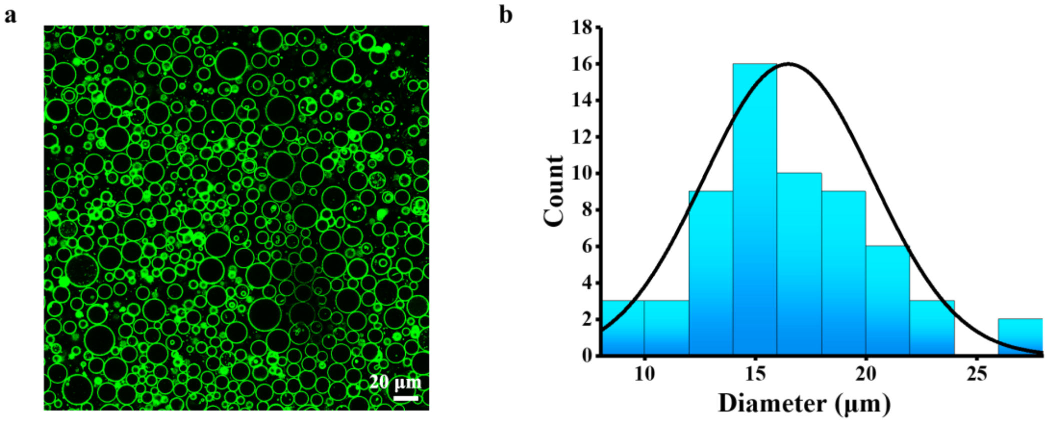

Supplementary Figure 7. Confocal microscopy image and diameter distribution of GUVs. a Confocal microscopy image of GUVs labeled with NBD-PE by emulsion method. **b** The diameter distribution of GUVs. N=141. The scale bar is 20 µm. Source data are provided as a Source Data file.

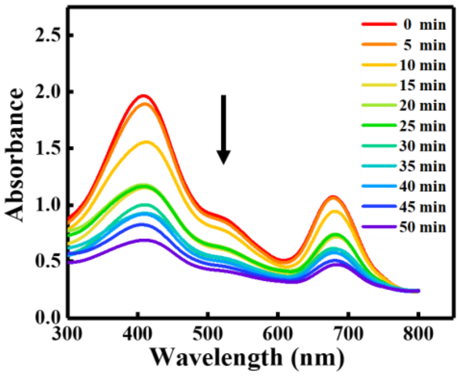

Supplementary Figure 8. UV absorption spectra of chlorin e6 (Ce6) treated with different irradiation time. The absorption peaks of 2 mM Ce6 under laser irradiation (405 nm) at different irradiation time (0 min, 5 min, 10 min, 15 min, 20 min, 25 min, 30 min, 35 min, 40 min, 45 min, 50 min). Source data are provided as a Source Data file.

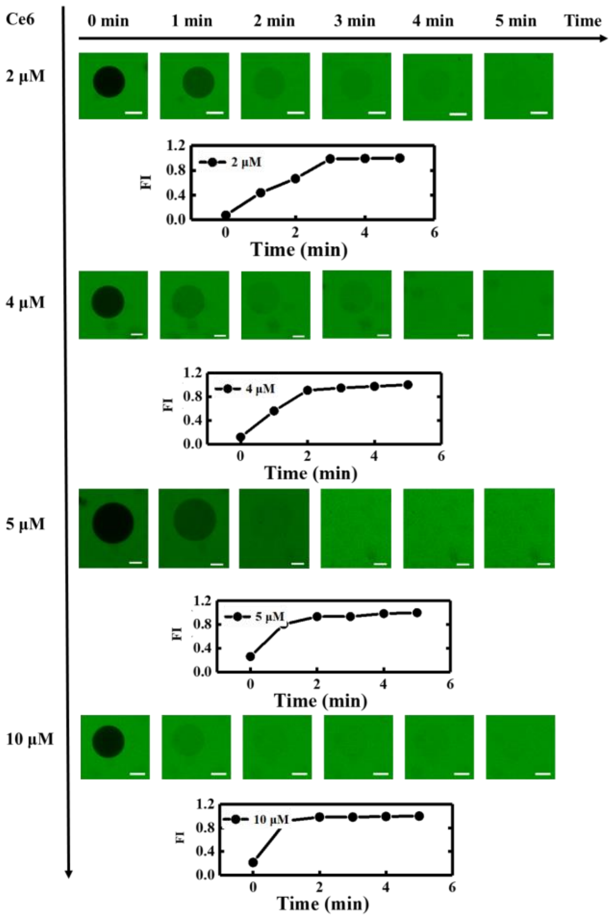

Supplementary Figure 9. The effect of chlorin e6 (Ce6) concentration on 8-Hydroxypyrene-1,3,6-trisulfonic acid, trisodium salt (HPTS) diffusion upon laser irradiation. Confocal microscopy images and normalized fluorescence intensity (FI) of HPTS inside GUVs against time at concentrations of Ce6 (2 μM, 4 μM, 5 μM, 10 μM) upon laser irradiation (405 nm, 0.3 mW, 5 s). The concentration of HPTS was 100 μM. The scale bars are 10 μm. Source data are provided as a Source Data file.

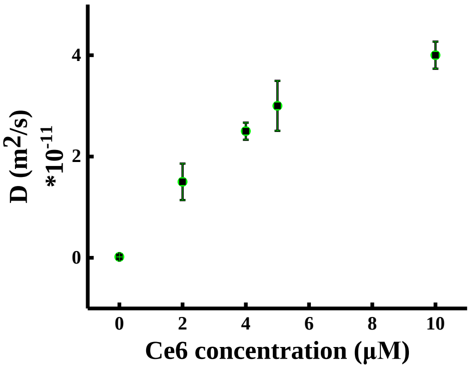

Supplementary Figure 10. The inflow diffusion coefficient (*D*) of 8-Hydroxypyrene-1,3,6-trisulfonic acid, trisodium salt (HPTS) at different chlorin e6 (Ce6) concentration. The *D* values were 1.5 × 10^{-11} m^2/s, 2.5 × 10^{-11} m^2/s, 3 × 10^{-11} m^2/s and 4 × 10^{-11} m^2/s at the Ce6 concentration of 2 μM, 4 μM, 5 μM, 10 μM, respectively. *D* was calculated according to the following formula 1:

$$\frac{I_A - I_B}{I_0} = \exp\left(-\frac{D}{A}t\right) \quad (1)$$

where I_A is the external mean fluorescence intensity of the GUV, I_B is the internal mean fluorescence intensity of the GUV, I_0 is the internal mean fluorescence intensity of the GUV at the beginning, and A is the surface area of GUV. Source data are provided as a Source Data file.

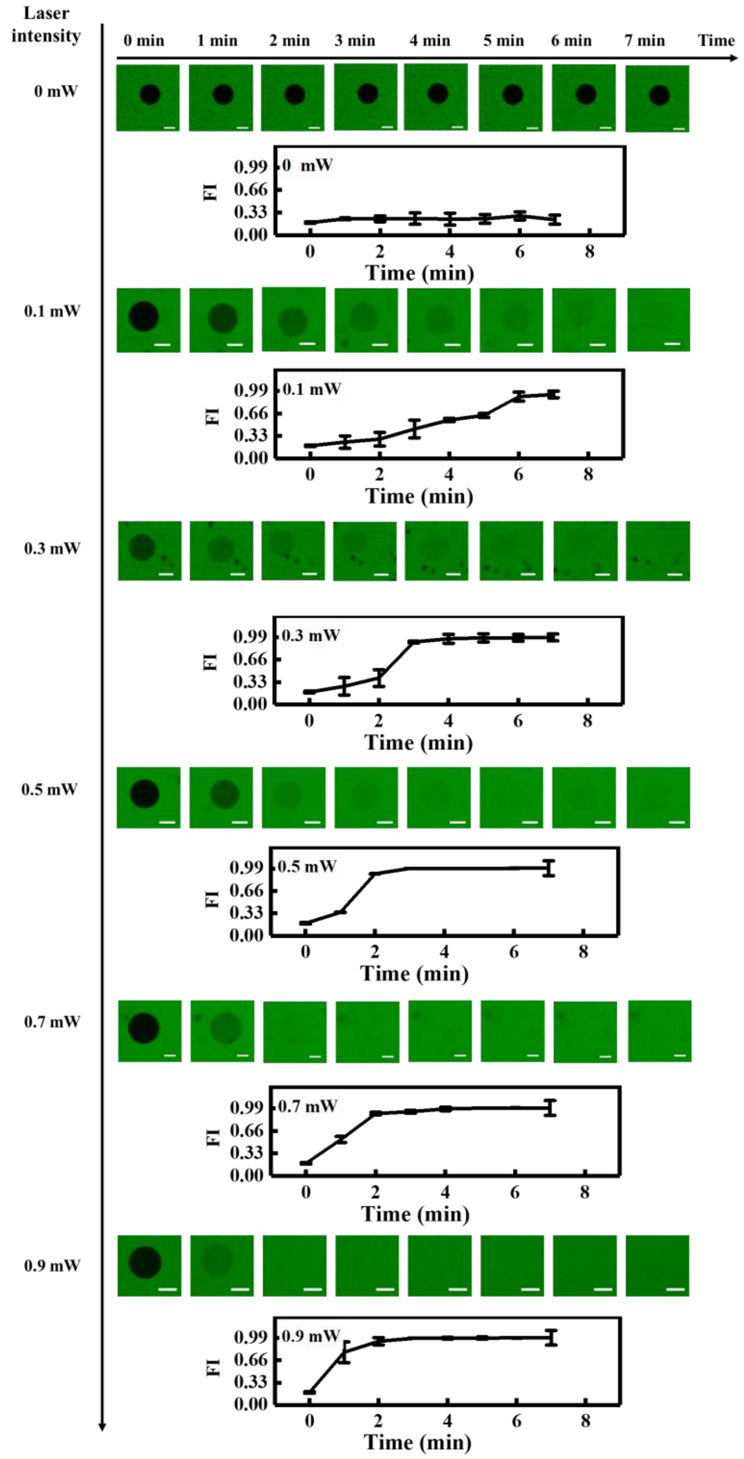

Supplementary Figure 11. The effect of laser intensities on 8-Hydroxypyrene-1,3,6-trisulfonic acid, trisodium salt (HPTS) inflow diffusion. Confocal microscopy images and normalized fluorescence intensity (FI) of GUVs in a solution of HPTS (100 μM) at the presence of Ce6 (10 μM) under different laser intensities (0 mW, 0.1 mW, 0.3 mW, 0.5 mW, 0.7 mW, and 0.9 mW) at 405 nm for 5 s. The scale bars are 10 μm. Source data are provided as a Source Data file.

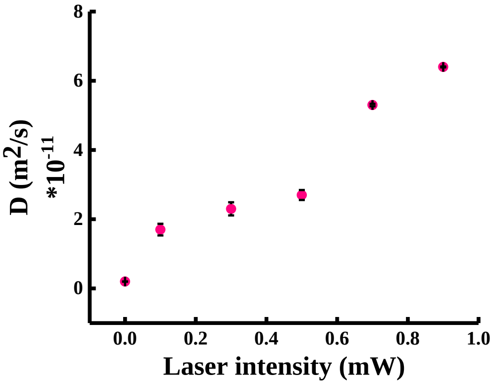

Supplementary Figure 12. The inflow diffusion coefficient (*D*) of 8-Hydroxypyrene-1,3,6-trisulfonic acid trisodium salt (HPTS) at different laser intensities. The *D* values were 0.2 × 10^{-11}, 1.7 × 10^{-11}, 2.3 × 10^{-11}, 2.7 × 10^{-11}, 5.3× 10^{-11}, and 6.4 × 10^{-11} m^2/s at laser intensity of 0.1, 0.3, 0.5, 0.7 and 0.9 mW, respectively. *D* was calculated according to the following formula 2:

$$\frac{I_A - I_B}{I_0} = \exp(-\frac{D}{A}t) \quad (2)$$

where I_A is the external mean fluorescence intensity of the GUV, I_B is the internal mean fluorescence intensity of the GUV, I_0 is the internal mean fluorescence intensity of the GUV at the beginning, and *A* is the surface area of GUV. Source data are provided as a Source Data file.

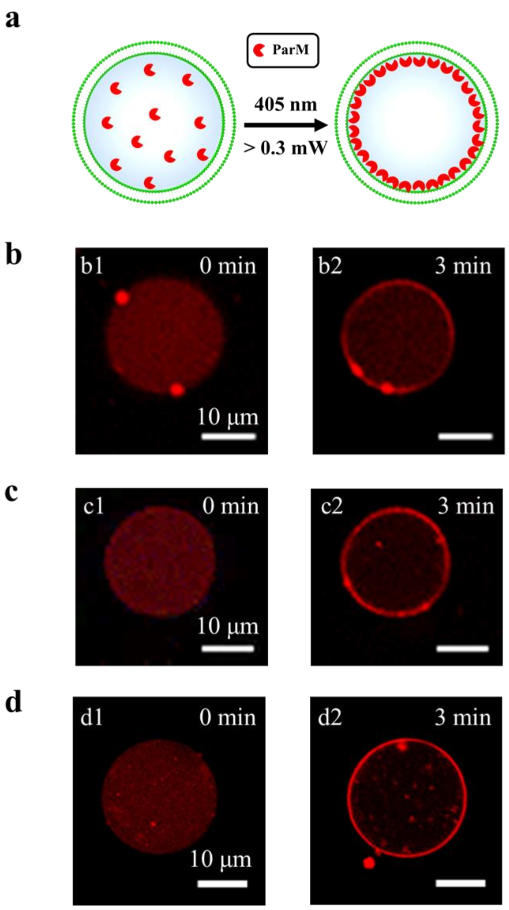

Supplementary Figure 13. The polymerization of ParM inside giant unilamellar vesicles (GUVs) at different laser intensities. a Schematic illustration of ParM polymerization inside GUVs upon the laser irradiation with power over 0.3 mW. Confocal microscopy images of ParM polymerization adjacent to the lipid bilayer of GUVs as a function of time upon 5 s laser (405 nm) irradiation with the power of 0.5 mW (**b**), 0.7 mW (**c**), and 0.9 mW (**d**), respectively. ParM concentration inside GUVs and ATP concentration outside GUVs are 9.6 μM and 1mM respectively. The scale bars are 10 μm.

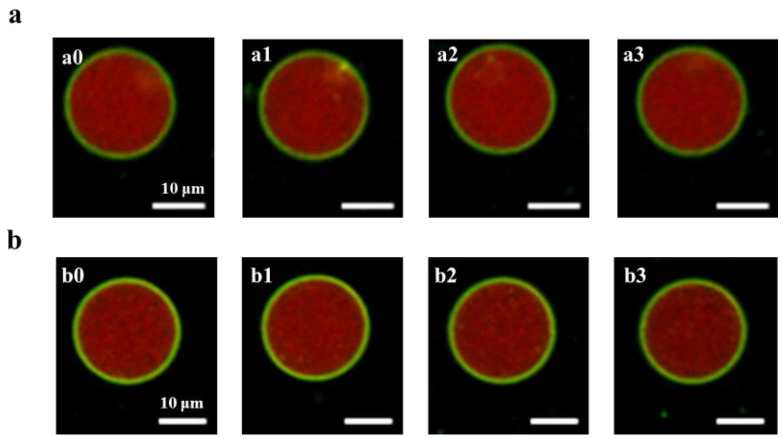

Supplementary Figure 14. The control experiments for Chlorin e6 (Ce6)-mediated adenosine triphosphate (ATP) influx. a Fluorescence images of GUVs (labelled with NBD-PE) containing 9.6 μM ParM upon laser irradiation (405 nm, 0.3 mW, 5 s) at the presence of 1 mM ATP with no addition of Ce6. **a0**, **a1**, **a2**, and **a3** were taken before irradiation and 0 min, 5 min and 10 min after irradiation, respectively. **b** Fluorescence images of GUVs (labelled with NBD-PE) containing 9.6 μM ParM in the presence of Ce6 (10 μM) and 1 mM ATP with no laser irradiation. **b0**, **b1**, **b2**, and **b3** were taken before the addition of Ce6 and ATP, and 0 min, 5 min and 10 min after the addition of Ce6, respectively. The scale bars are 10 μm.

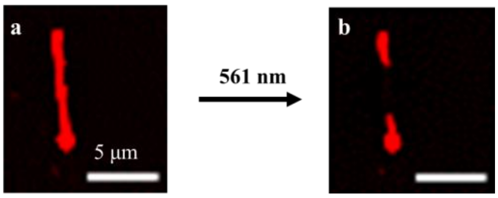

Supplementary Figure 15. The split of a ParM filament in solution by laser irradiation. Fluorescence images of the ParM filament before (**a**) and after (**b**) laser irradiation (561 nm, 0.7 mW, 5 s). The scale bars are 5 µm.

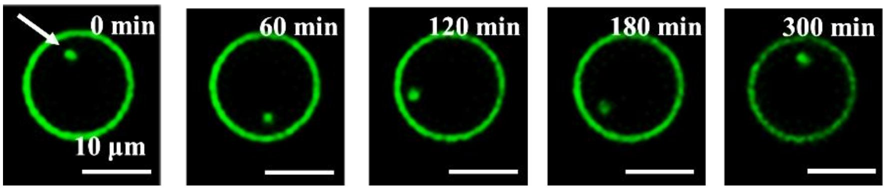

Supplementary Figure 16. Confocal microscopy images of the giant unilamellar vesicles (GUV) containing *parC*-bead. Time-dependent fluorescence images of the GUV containing the *parC*-bead after the addition of Ce6 (10 μM) and laser irradiation (405 nm, 0.3 mW, 5 s) at 0 min, 60 min, 120 min, 180 min, 300 min, respectively. The scale bars are 10 μm.

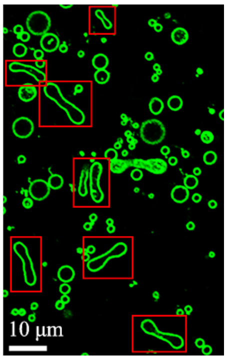

Supplementary Figure 17. Confocal microscopy images of deformed giant unilamellar vesicles (GUV). The deformation of GUVs was triggered by the osmotic pressure caused by 70 mM difference between outside (330 mM glucose) and inside GUVs (260 mM sucrose). GUVs membranes were labeled with NBD-PE (green fluorescence). The GUVs in the red rectangles are deformed GUVs (dumbbell shape GUVs). The scale bar is 10 µm.

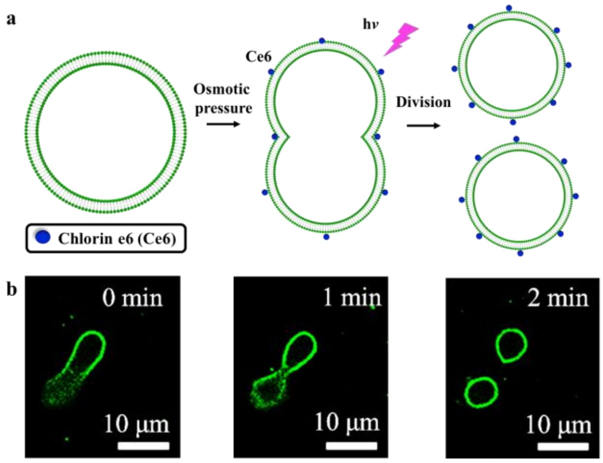

Supplementary Figure 18. Schematic illustration and confocal microscopy images of the giant unilamellar vesicles (GUV) division. a Schematic diagram of GUVs division. **b** Confocal time series of GUV division at hypertonic condition (△c = 70 mM), followed by Ce6-mediated the GUV division due to peroxidation of unsaturated lipid POPC upon laser irradiation (405 nm, 0.3 mW, 5 s). GUVs membranes were labeled with NBD-PE (green fluorescence). The scale bars are 10 μm.

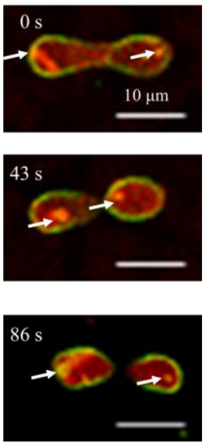

Supplementary Figure 19. The confocal time series images of *parC*-beads redistribution through giant unilamellar vesicles (GUV) division. The *parC*-beads positioned to two poles of GUV by the split of ParM filament, and redistributed into two daughter GUVs after division. GUV membrane was labeled with NBD-PE (green fluorescence). ParM filament labeled with cy3 (red fluorescence). *parC*-beads labeled with SYBR Green I (green fluorescence). The white arrows referred to the *parC*-beads. The scale bars are 10 μm.

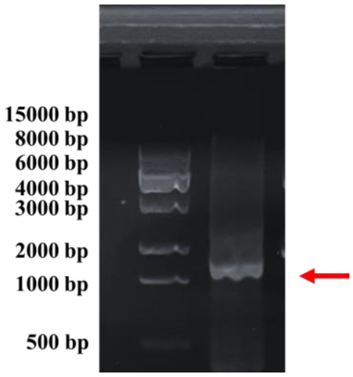

Supplementary Figure 20. Validation of the amplified products of *parC*-eGFP DNA by agarose gel. Agarose gel of the amplified biotinylated *parC*-eGFP DNA. The red arrow indicated the band of amplified products (~1000 bp). Source data are provided as a Source Data file.

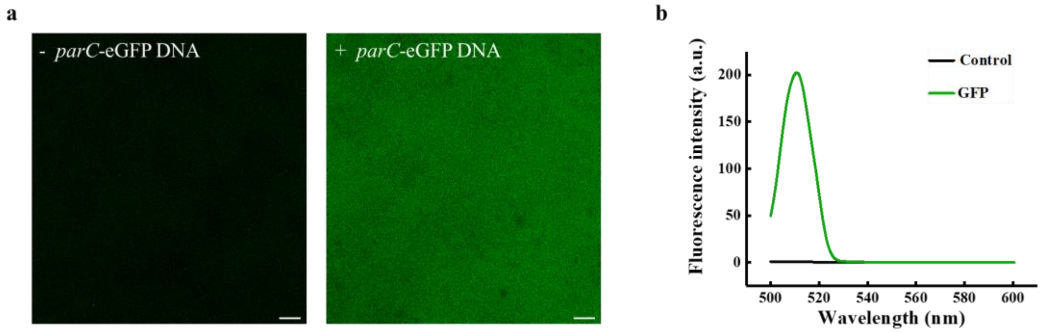

Supplementary Figure 21. The expression of enhanced green fluorescent protein (eGFP) using the PURE system in solution. a Confocal microscopy images of eGFP expression using the protein synthesis using recombinant elements (PURE system) in solution. 10 µL of PUREfrex 2.1 systems contained 4 µL solution I (amino acids, NTPs, tRNAs, enzyme substrates, etc.), 0.5 µL of solution II (proteins), 1 µL of solution III (20 µM ribosomes), 1 µL of cysteine (3 mM), 0.5 µL of GSH (80 mM), 0.5 µL of the plasmid encoding for eGFP (20 ng/µL), and 2.5 µL of nuclease-free water. **b** The fluorescence spectra of eGFP solution. The scale bars are 20 µm. Source data are provided as a Source Data file.

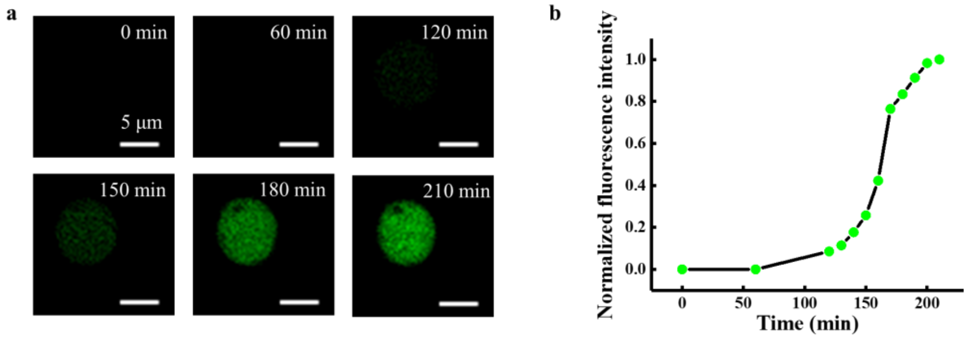

Supplementary Figure 22. Confocal microscopy images and fluorescence intensities of eGFP expressed inside GUV as a function of time. a Confocal microscopy images of the expression of eGFP inside a GUV as a function of time at 37°C. The solution inside GUV was PUREfrex 2.1 systems. The scale bars are 5 μm. **b** The normalized fluorescence intensities of expressed eGFP inside GUV as a function of time corresponding to Supplementary Figure S**22a**. Source data are provided as a Source Data file.

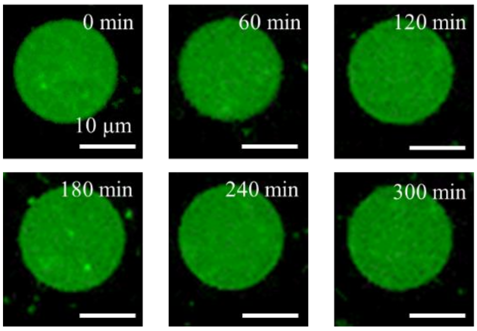

Supplementary Figure 23. Confocal microscopy images of the GUV containing eGFP. Time-dependent fluorescence images of eGFP inside the GUV after the addition of Ce6 (10 μM) and laser irradiation (405 nm, 0.3 mW, 5 s) at 0 min, 60 min, 120 min, 180 min, 240 min, 300 min, respectively. The scale bars are 10 μm.

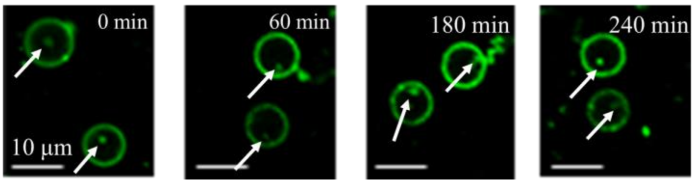

Supplementary Figure 24. Confocal microscopy images of two daughter cells without PURE system. No enhance green fluorescence proteins (eGFP) were detected inside GUV at 37°C for 3 hours due to the absence of PURE system. GUV membranes were labeled with NBD-PE (green fluorescence). *parC*-beads were labeled with SYBR Green I (green fluorescence). The white arrows referred to the *parC*-beads. The scale bars are 10 µm.

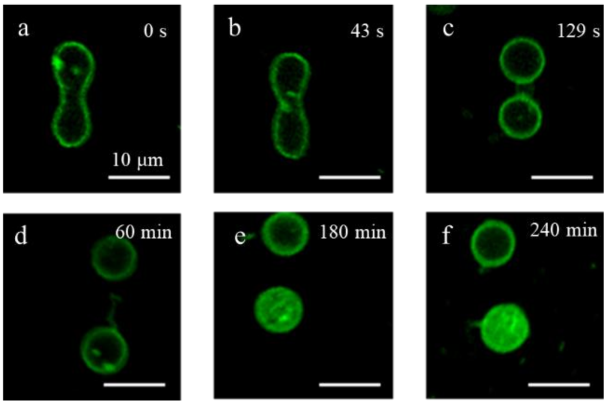

Supplementary Figure 25. Confocal microscopy images of GUV division and follow-on eGFP expression in two daughter GUVs without ParMRC system. Time-dependent fluorescence images of giant unilamellar vesicles (GUV) division at 0 s (**a**), 43 s (**b**), 129 s (**c**), and enhanced green fluorescent protein (eGFP) expression at 37 °C in daughter cells at 60 min (**d**), 180 min (**e**), 240 min (**f**), respectively. The scale bars are 10 μm.

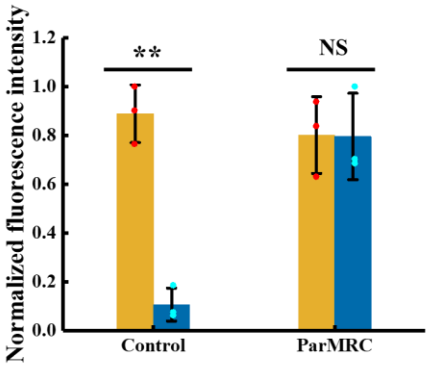

Supplementary Figure 26. The normalized fluorescence intensity of eGFP in daughter GUV 1 and GUV 2 with or without ParMRC system. The control experiments represent the condition of enhanced green fluorescent protein (eGFP) expression in daughter GUV 1 and GUV 2 without the ParMRC system. The normalized fluorescence intensities of eGFP are from three independent samples. Data are presented as the mean values ± SDs, n=3. Statistical analyses were carried out by unpaired two-tailed student's t-test. **p=0.000578, Degrees of freedom=4, effect size statistic=0.07877, Confidence Intervals=95%. The ParMRC experiments represent the condition of eGFP expression in daughter GUV 1 and GUV 2 with the ParMRC system. The normalized fluorescence intensities of eGFP are from three independent samples. Data are presented as the mean values ± SDs, n=3. Statistical analyses were carried out by unpaired two-tailed student's t-test. NS, not significant (P=0.9670), Degrees of freedom=4, effect size statistic=0.1367, Confidence Intervals=95%. Source data are provided as a Source Data file.

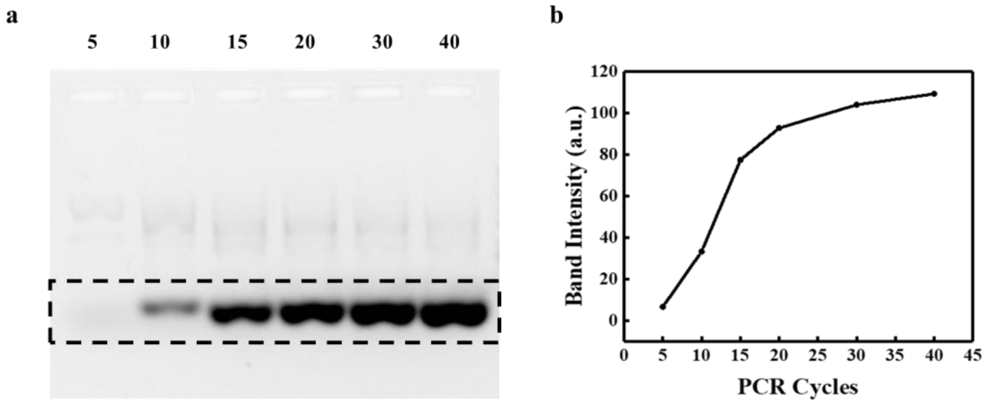

Supplementary Figure 27. Validation of the amplified biotinylated *parC* by agarose gel. a Agarose gel image of amplified the biotinylated *parC* after 5, 10, 15, 20, 30, and 40 thermal cycles. The bands in the black box referred to the amplified biotinylated *parC*. **b** The corresponding intensity of the bands in the black box in Supplementary Figure **27a**. Source data are provided as a Source Data file.

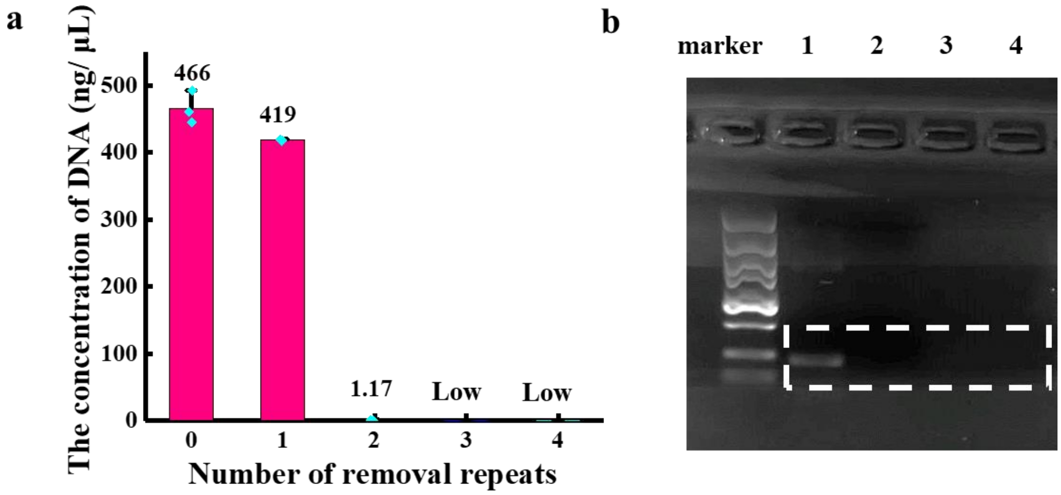

Supplementary Figure 28. Removal of redundant *parC* DNA. a The concentrations of DNA in the supernatants of the buffer solution as a function of removal repeats (0-4). **b** Corresponding agarose gel image of the supernatants after 1, 2, 3, and 4 removal cycles in Supplementary Figure **28a**. The white box indicated the band of remaining DNA. Source data are provided as a Source Data file.

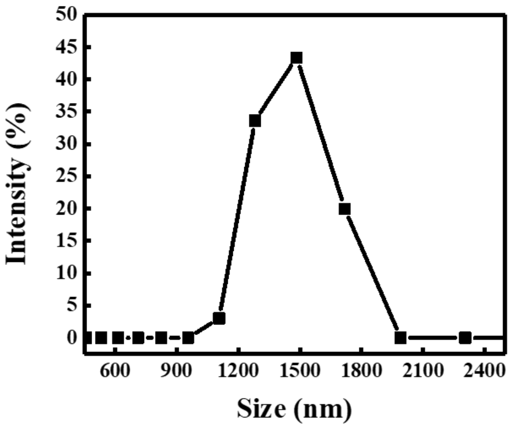

Supplementary Figure 29. Dynamic light scattering (DLS) data of the biotinylated *parC*-beads. The size of *parC*-beads was 1352 ± 206 nm. The solution contained 260 mM sucrose, 30 mM Tris-HCl, 2 mM $MgCl_2$, 1 mM DTT, 100 mM KCl at pH 7.5. Source data are provided as a Source Data file.

Supplementary Figure 20. The uncropped scans of agarose gel of *parC*-eGFP DNA.

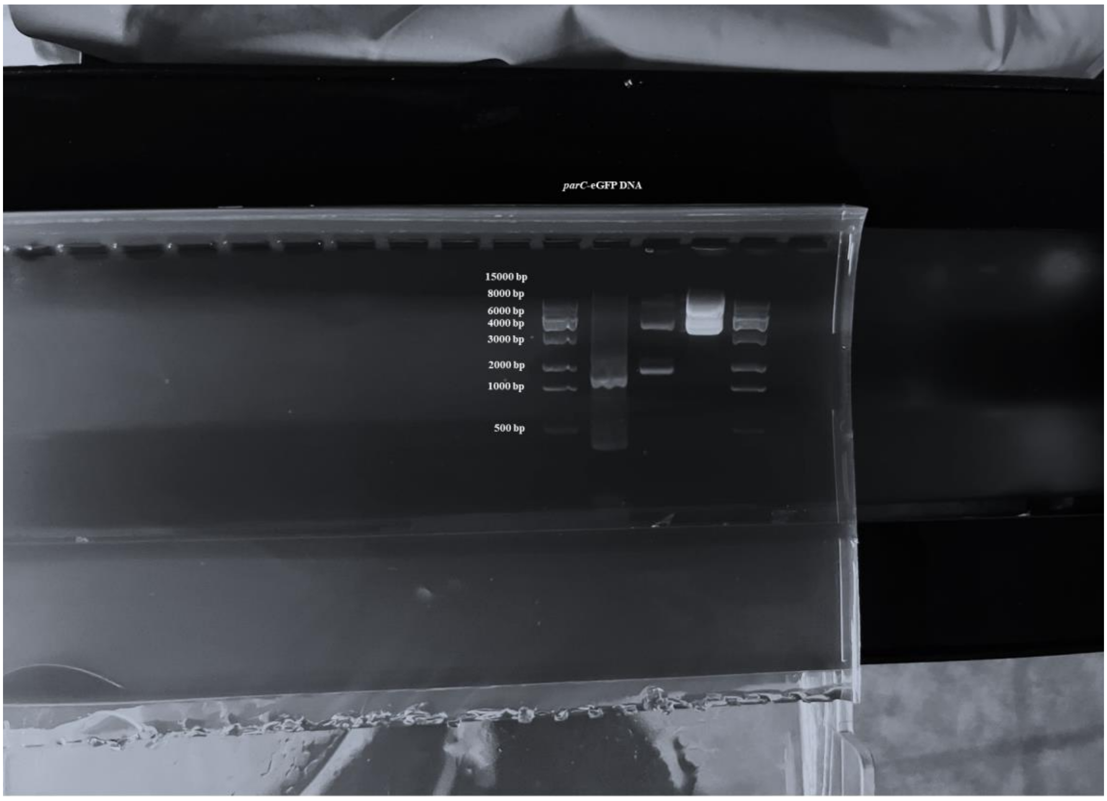

Supplementary Figure 27a. The uncropped scans of agarose gel of amplified biotinylated *parC*.

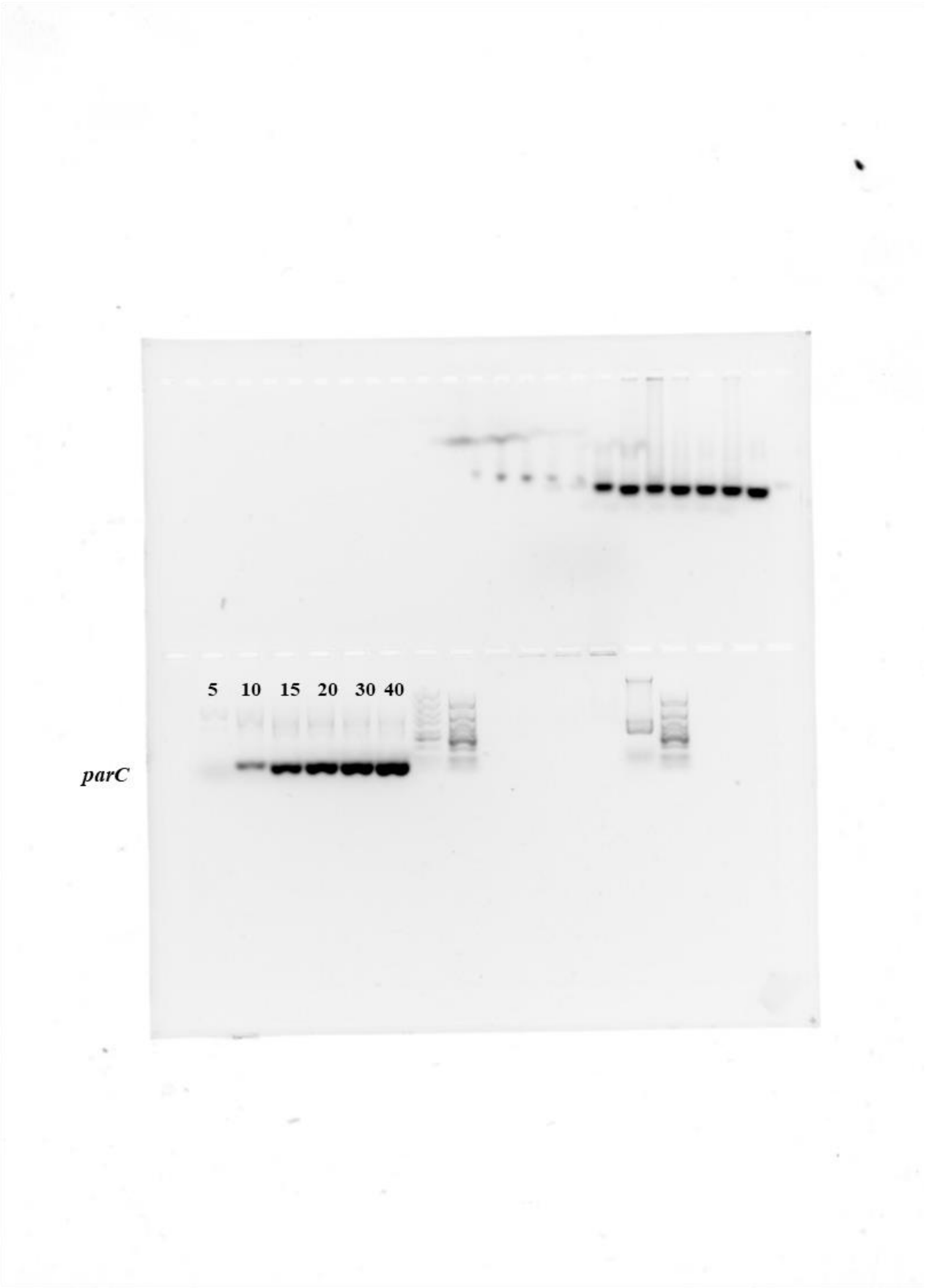

Supplementary Figure 28b. The uncropped scans of agarose gel of removal of redundant *parC* DNA.

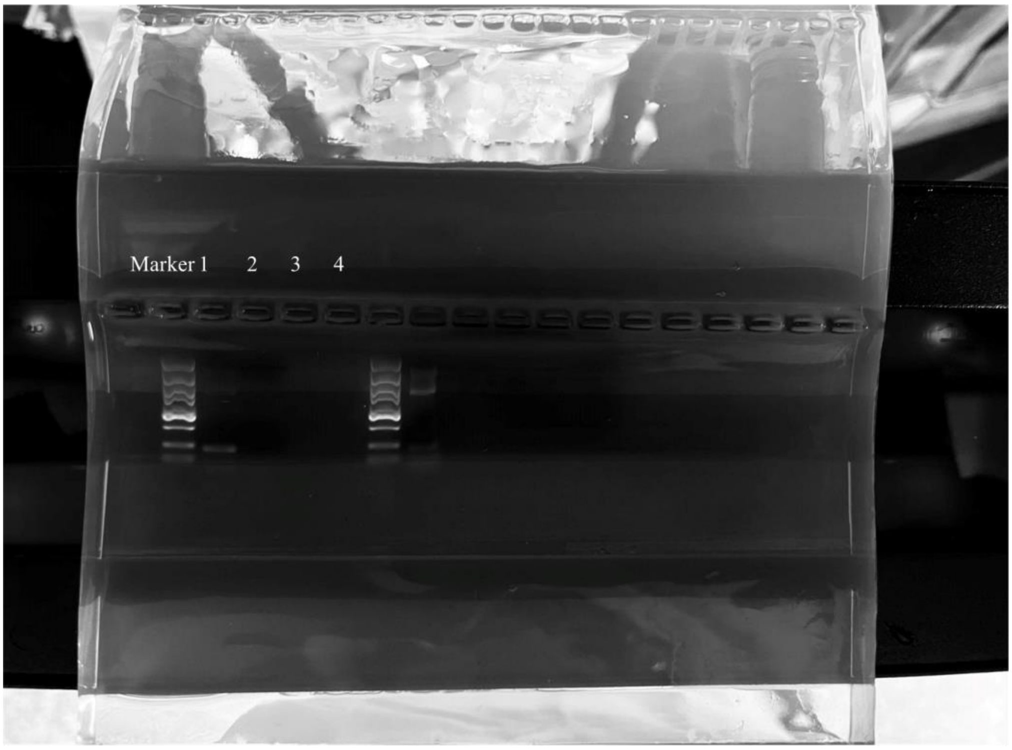

Supplement: Supplementary file 1 — Supplemental Information [file 41467_2024_49412_MOESM1_ESM.pdf]
