# Supplementary material for: Investigation of artificial cells containing the Par system for bacterial plasmid segregation and inheritance mimicry
Source: Nat Commun. 2024 Jun 10;15:4956. doi: 10.1038/s41467-024-49412-9 (PMC11164925; doi:10.1038/s41467-024-49412-9)
Supplement: Supplementary file 2 — Reporting Summary [file 41467_2024_49412_MOESM2_ESM.pdf]

nature portfolio

Corresponding author(s): Xiaojun Han

Last updated by author(s): May 31, 2024

Reporting Summary

Nature Portfolio wishes to improve the reproducibility of the work that we publish. This form provides structure for consistency and transparency in reporting. For further information on Nature Portfolio policies, see our Editorial Policies and the Editorial Policy Checklist.

Statistics

For all statistical analyses, confirm that the following items are present in the figure legend, table legend, main text, or Methods section.

n/a	Confirmed	
☐	☒	The exact sample size (*n*) for each experimental group/condition, given as a discrete number and unit of measurement
☐	☒	A statement on whether measurements were taken from distinct samples or whether the same sample was measured repeatedly
☐	☒	The statistical test(s) used AND whether they are one- or two-sided<br>*Only common tests should be described solely by name; describe more complex techniques in the Methods section.*
☒	☐	A description of all covariates tested
☒	☐	A description of any assumptions or corrections, such as tests of normality and adjustment for multiple comparisons
☐	☒	A full description of the statistical parameters including central tendency (e.g. means) or other basic estimates (e.g. regression coefficient) AND variation (e.g. standard deviation) or associated estimates of uncertainty (e.g. confidence intervals)
☐	☒	For null hypothesis testing, the test statistic (e.g. *F*, *t*, *r*) with confidence intervals, effect sizes, degrees of freedom and *P* value noted<br>*Give P values as exact values whenever suitable.*
☒	☐	For Bayesian analysis, information on the choice of priors and Markov chain Monte Carlo settings
☒	☐	For hierarchical and complex designs, identification of the appropriate level for tests and full reporting of outcomes
☒	☐	Estimates of effect sizes (e.g. Cohen's *d*, Pearson's *r*), indicating how they were calculated

Our web collection on statistics for biologists contains articles on many of the points above.

Software and code

Policy information about availability of computer code

Data collection	Gel imaging instrument (Amersham Imager 600, GE) was used to detect the bands of extracted ParM, ParR and parC. A microplate reader (Molecular Devices, SpectraMax iD3, Germany) was used to test protein concentration and phosphate release. Circular dichroism instrument (Chirascan, Applied Photophysics, UK) was used to analyze the secondary structure of ParR and ParR-parC. A fluorescence microscope (Olympus IX73, Japan) and a confocal laser scanning microscope (Olympus FV 3000, Japan) were used to obtain all fluorescence images. UV-Vis spectrometer (Agilent Technologies Cary 60) was used to measure the concentration of ATP. Fluorescence spectrometer (PerkinElmer Fluorescence spectrometer LS 55) was used to detect the fluorescence emission spectrum curve of eGFP synthesized in vitro. Automated cell counter (Countess 3, Thermo Fisher Scientific) was used to measure the concentration of streptavidin beads.
Data analysis	Fluorescence microscope images were analyzed by CellSens21, and Image J. Data process was conducted using Origin 2017, GraphPad Prism 9 , and Office2019.

For manuscripts utilizing custom algorithms or software that are central to the research but not yet described in published literature, software must be made available to editors and reviewers. We strongly encourage code deposition in a community repository (e.g. GitHub). See the Nature Portfolio guidelines for submitting code & software for further information.

Data

Policy information about availability of data

All manuscripts must include a data availability statement. This statement should provide the following information, where applicable:

- Accession codes, unique identifiers, or web links for publicly available datasets
- A description of any restrictions on data availability
- For clinical datasets or third party data, please ensure that the statement adheres to our policy

The authors declare that all data generated in this study are provided in the Supplementary Information/Source Data file. The source data underlying Fig. 2a-h,3b, 3e, 4d, 5d, 5e and Supplementary Fig. 3, 7, 8, 9, 10, 11, 12, 20,21, 22, 26, 27a-b, 28a-b, 29 are provided as a Source Data file.

Research involving human participants, their data, or biological material

Policy information about studies with human participants or human data. See also policy information about sex, gender (identity/presentation), and sexual orientation and race, ethnicity and racism.

Reporting on sex and gender	N/A
Reporting on race, ethnicity, or other socially relevant groupings	N/A
Population characteristics	N/A
Recruitment	N/A
Ethics oversight	N/A

Note that full information on the approval of the study protocol must also be provided in the manuscript.

Field-specific reporting

Please select the one below that is the best fit for your research. If you are not sure, read the appropriate sections before making your selection.

☒ Life sciences ☐ Behavioural & social sciences ☐ Ecological, evolutionary & environmental sciences

For a reference copy of the document with all sections, see nature.com/documents/nr-reporting-summary-flat.pdf

Life sciences study design

All studies must disclose on these points even when the disclosure is negative.

Sample size	The gel images of ParM, ParR,parC, parC-beads,and EMSA of parC with increasing concentrations of ParR were repeated three times.The experiments of phosphate release (Pi) were tested for three times. The sample sizes of ParM filaments lengths were 100. The sample size of GUVs was 141.The sample sizes of the growth of ParM filaments inside lipid protected droplets and GUVs were 3. A ParM filament was splitted inside the GUV were tested for three times. Two parC-beads segregated by ParM filament inside the GUV were tested for three times. GUV division and eGFP expression in daughter cells with ParMRC system were tested for three times. eGFP in daughter GUV 1 and GUV 2 without ParMRC system were tested for three times. Removal of redundant parC DNA were tested for three times.
Data exclusions	No such exclusion in this paper.
Replication	The experiments were independently replicated at least three times. The reproducible results were always obtained.
Randomization	No randomization was used.
Blinding	No blinding was necessary for this study.

Reporting for specific materials, systems and methods

We require information from authors about some types of materials, experimental systems and methods used in many studies. Here, indicate whether each material, system or method listed is relevant to your study. If you are not sure if a list item applies to your research, read the appropriate section before selecting a response.

Materials & experimental systems

n/a	Involved in the study	
☒	☐	Antibodies
☒	☐	Eukaryotic cell lines
☒	☐	Palaeontology and archaeology
☒	☐	Animals and other organisms
☒	☐	Clinical data
☒	☐	Dual use research of concern
☒	☐	Plants

Methods

n/a	Involved in the study	
☒	☐	ChIP-seq
☒	☐	Flow cytometry
☒	☐	MRI-based neuroimaging

Plants

Seed stocks	N/A
Novel plant genotypes	N/A
Authentication	N/A
