# Supplementary material for: Investigation of artificial cells containing the Par system for bacterial plasmid segregation and inheritance mimicry
Source: Nat Commun. 2024 Jun 10;15:4956. doi: 10.1038/s41467-024-49412-9 (PMC11164925; doi:10.1038/s41467-024-49412-9)
Supplement: Supplementary file 3 — Description of Additional Supplementary Files [file 41467_2024_49412_MOESM3_ESM.pdf]

Supplementary Movie 1.
The growth of a ParM filament as a function of time inside the lipid-protected droplet.

Supplementary Movie 2.
The growth of a ParM filament as a function of time inside the giant unilamellar vesicles.

Supplementary Data 1:
Sequences of gene of ParM, ParR, *parC*, eGFP, primer 1, primer 1, primer1' and primer 2' used in current study.
